# Oral Factors That Impact the Oral Microbiota in Parkinson’s Disease

**DOI:** 10.3390/microorganisms9081616

**Published:** 2021-07-29

**Authors:** Natalia S. Rozas, Gena D. Tribble, Cameron B. Jeter

**Affiliations:** 1 Department of Diagnostic and Biomedical Sciences, School of Dentistry, The University of Texas Health Science Center at Houston (UTHealth), 7500 Cambridge St., Suite 5371, Houston, TX 77054, USA; Natalia.S.Rozas@uth.tmc.edu; 2 Department of Periodontics and Dental Hygiene, School of Dentistry, The University of Texas Health Science Center at Houston (UTHealth), 1941 East Road, BBS-5318, Houston, TX 77054, USA; Gena.D.Tribble@uth.tmc.edu

**Keywords:** microbiota, oral health, oral hygiene, periodontitis, periodontal disease, aspiration pneumonia

## Abstract

Patients with Parkinson’s disease (PD) are at increased risk of aspiration pneumonia, their primary cause of death. Their oral microbiota differs from healthy controls, exacerbating this risk. Our goal was to explore if poor oral health, poor oral hygiene, and dysphagia status affect the oral microbiota composition of these patients. In this cross-sectional case-control study, the oral microbiota from hard and soft tissues of patients with PD (*n* = 30) and age-, gender-, and education-matched healthy controls (*n* = 30) was compared using 16S rRNA gene sequencing for bacterial identification. Study participants completed dietary, oral hygiene, drooling, and dysphagia questionnaires, and an oral health screening. Significant differences in soft tissue beta-diversity (*p* < 0.005) were found, and a higher abundance of opportunistic oral pathogens was detected in patients with PD. Factors that significantly influenced soft tissue beta-diversity and microbiota composition include dysphagia, drooling (both *p* < 0.05), and salivary pH (*p* < 0.005). Thus, patients with PD show significant differences in their oral microbiota compared to the controls, which may be due, in part, to dysphagia, drooling, and salivary pH. Understanding factors that alter their oral microbiota could lead to the development of diagnostic and treatment strategies that improve the quality of life and survivability of these patients.

## 1. Introduction

Parkinson’s disease (PD) is the fastest growing neurological condition in prevalence, disability, and deaths [1]. PD is categorized as a movement disorder due to its well-known motor symptoms of bradykinesia, resting tremor, and rigidity. Regrettably, PD patients also suffer from a variety of other debilitating symptoms such as dysarthria (difficulty speaking), dysphagia (difficulty swallowing), and sialorrhea (drooling) [2]. These oral symptoms, especially in conjunction with memory loss and depression, may lead to malnutrition, social isolation, and oral health problems [3,4].

Poor oral health in patients with PD has been documented repeatedly and includes more missing teeth, dental plaque, dental caries, and bone loss [5,6,7]. Microorganisms grow as biofilms on both the hard tissues (teeth) and soft tissues (e.g., tongue, gums, or inner cheek) of the oral cavity, and their composition differs by oral location [8]. While the species of bacteria in oral biofilms are commensal and symbiotic with the host during health, they can shift to opportunistic and hostile depending on the virulence of the bacteria, host immunological response, and environmental factors such as smoking or frequency of oral hygiene [8]. The oral microbiota of patients with PD differs from that of age- and gender-matched controls, but it is unknown what factors underpin this difference [9,10,11].

Oral motility serves to move food and debris from the mouth and is the body’s natural defense mechanism against oral infections and tooth decay. Restriction of oral movement in persons with PD leaves them highly prone to accumulation of oral pathogens, infection, and inflammation [4,12]. In addition, dysphagia can result in entrance of food, drink, or saliva into the air passages and lead to aspiration pneumonia, a bacterial infection of the lungs or large airways [13]. Indeed, aspiration pneumonia afflicts half of patients with PD (three times the incidence in age-matched controls) and is their leading cause of mortality [14,15,16]. Understanding how oral dysbiosis develops is necessary to design strategies to restore a healthy state, reduce oral infections, and prevent death by aspiration pneumonia in these patients [17].

Our objectives were to (1) further characterize the oral microbiota in PD and (2) identify factors of oral health and oral symptoms that may explain its difference from that of age, gender-, and education-matched controls. Our results show significant differences in soft tissue beta-diversity and a higher abundance of opportunistic oral pathogens in patients with PD. Factors that significantly influenced soft tissue beta-diversity and microbiota composition include dysphagia, drooling, and salivary pH.

## 2. Materials and Methods

### 2.1. Subject Population and Study Design

The study was a cross-sectional case-control design that examined the oral (hard and soft tissue) microbiota through 16S rRNA gene sequencing of patients with PD and healthy controls. The study was approved by The University of Texas Health Science Center at Houston (UTHealth) Committee for the Protection of Human Subjects (HSC-DB-14-0382, first approved 23 May 2014), and all participants gave informed consent. Patients were recruited from the movement disorders clinics of UT Physicians, the clinical arm of the UTHealth McGovern Medical School, as well as the UTHealth School of Dentistry and Quentin Mease Community Hospital of the Harris Health System. Inclusion criteria were: (1) diagnosis of a parkinsonian syndrome by a neurologist, and (2) age 50 years or older. Exclusion criteria were: (1) neurological deficit prior to onset of movement disorder (e.g., previous stroke, dementia), (2) history of tobacco use within the last 10 years, and (3) antibiotic use in the last three months. The controls were enrolled from the community by word of mouth and from Settegast and Baytown Community Clinics of the Harris Health System and the UTHealth School of Dentistry. The latter participants were seeking routine prophylaxis (cleanings) or periodontal maintenance. Inclusion criteria for the controls were: (1) no significant medical history, (2) no significant neurological or psychiatric disorders, (3) no history of tobacco use within the last 10 years, and (4) no antibiotic use in the last three months.

All participants were recruited between June 2014 and December 2015. Data and samples were collected at time of recruitment. To reduce bias, all patients attending the above clinics on the days of enrollment were asked to participate in the study; those that accepted and qualified were enrolled.

### 2.2. Oral Health and Demographics Data Collection

Participants completed four standardized structured questionnaires (general health, oral hygiene habits, dietary habits, and saliva and swallowing subscales of the Radboud Oral Motor Inventory (ROMP)), and a trained dental student completed an oral health screening comprised of the Brief Oral Health Status Examination (BOHSE) and the Simplified Oral Hygiene Index (OHI-S) [18,19,20,21]. The OHI-S includes quantification of tooth plaque before and after participants brush their teeth. In this way, the OHI-S also evaluates participants’ effectiveness in toothbrushing. Saliva pH was measured with pH paper indicator dipped in a saliva sample.

### 2.3. Microbiota Sample Collection

Two oral microbiota samples were collected from each participant: hard tissues (lingual sides of the lower incisors and chewing surfaces of all lower right molars) and soft tissues (tongue dorsum and buccal mucosa). Oral sites were sampled using Catch-All™ Sample Collection Swabs (Epicentre Technologies, Madison, WI, USA), immediately transferred to separate MO BIO PowerBead tubes (Qiagen, Germantown, MD, USA) and kept cold until DNA extraction.

### 2.4. Bacterial Community Sequencing and Analysis

Bacterial genomic DNA was extracted from specimens using the MO BIO PowerSoil DNA Isolation Kit (MO BIO Laboratories, Carlsbad, CA, USA) and stored at −80 °C until analysis. The 16S rRNA V4 region was PCR amplified and sequenced on the Illumina MiSeq platform using a 2  ×  250-bp paired-end protocol adapted from the Human Microbiome Project methods [22]. Amplification primers contained adapters for MiSeq sequencing and single-index barcodes, resulting in PCR products that were pooled and sequenced directly. Read pairs were de-multiplexed based on barcodes and merged using CLC Genomics Workbench v10, with the Microbial Genomics module. 16S rRNA gene sequences were allocated to specific operational taxonomic units (OTUs) at 98% identity within the Human Oral Microbiome Database (HOMD) [23]. The HOMD library is manually curated by experts in oral microbiology and represents 529 taxa arranged in the taxonomic hierarchy. The HOMD taxa are defined to the species level. Using this reference library in CLC Genomics Workbench, we compared our sequences at 98%, and allowed taxonomic assignment from the HOMD library. If the OTU did not match 98% to a specific sequence, it was then assigned the appropriate taxonomic designation within the species/genus/family.

Community alpha and beta diversities were assessed using the Microbial Genomics module of CLC Genomics Workbench. OTUs from the abundance table were aligned using MUSCLE with a required minimum abundance of 10. Aligned OTUs were used to construct a phylogenetic tree using Maximum Likelihood Phylogeny using the Neighbor Joining method and the Jukes Cantor substitution model. Rarefication analysis was achieved by sub-sampling the OTU abundances in the different samples at a range of depths from 1 to 100,000; the number of different depths sampled was 20, with 100 replicates at each depth. Alpha-diversity measures were calculated for observed OTUs using Chao 1-bias corrected, Shannon entropy, and Simpsons Index. Statistical significance in alpha-diversity between cohorts was calculated with one-way ANOVA and post hoc tests by Bonferroni. Permutational Multivariate Analysis of Variance (PERMANOVA) analysis was used to detect significant differences in beta-diversity between groups, and comparisons were visualized using Principal Coordinate Analysis. Beta-diversity measures were calculated using Bray-Curtis, Jaccard, and Unweighted and Weighted UniFrac formulas. Differential abundance tests (non-parametric ANOVA) on the OTU frequency table were used to identify significant differences in the relative abundances of individual OTUs between groups. Differential abundance analysis values were calculated for the max group means (maximum of the average reads per kilobase of transcript, per million mapped reads (RPKM)), -log_2_ fold change, fold change, standard *p*-value (significance at less than 0.05), and FDR *p*-value (false discovery rate corrected *p*-value).

### 2.5. Statistical Analysis for Demographics and Oral Health

Results are expressed as mean, standard deviation (SD) or percentage (%). Student’s *t*-test was used to assess differences in continuous demographics, oral health, and oral hygiene data between groups. Chi-square (χ2) test was used to assess the differences in categorical demographics, diet, and oral hygiene data between groups. A *p*-value of less than 0.05 was considered statistically significant.

## 3. Results

### 3.1. Subject Characteristics

There were no significant differences in gender, age, ethnicity, race, education, or BMI between patients with PD and the controls (Table 1). About 70% of patients with PD took antiparkinsonian medications, and over 50% of these took more than one type of PD medication. A history of thyroid problems and consumption of aspirin was more prevalent in the controls (Table 1).

General oral health measured by BOHSE was statistically worse in patients with PD than in controls (Student’s *t*-test, *p* = 0.02) (Table 2). As measured by OHI-S, patients with PD had greater accumulation of tooth plaque than the controls (Student’s *t*-test, *p* = 0.006). After brushing their teeth, however, their levels were comparable to the controls’ plaque after brushing, representing a significant removal of plaque (Students *t*-test, *p* = 0.01). On the ROMP questionnaires, patients with PD reported significantly greater drooling problems and difficulty swallowing than the controls (Student’s *t*-test, each *p* < 0.001). Salivary pH did not significantly differ between the two groups, but trended toward more acidic in patients. There were no significant differences in general oral hygiene habits (tooth brushing, flossing, and dental visits) between patients with PD and the controls. When asked about their dietary habits, patients with PD reported consuming more snacks per day than the controls, and eating more foods with added sugar (Student’s *t*-test, each *p* = 0.02) (Table 2).

### 3.2. Microbiota Diversity

For our initial comparisons of bacterial community composition, we calculated alpha-diversity for all samples, and grouped the data into four cohorts by hard tissue and soft tissue, for both patients with PD and the controls (Figure S1). Alpha-diversity is an estimation of species richness and evenness within a group. The average number of OTUs in the hard tissue samples was 96 and 92 for patients with PD and the controls, respectively. The soft tissue values were 92 and 88 OTUs, respectively. Statistical comparisons of alpha-diversity between patients with PD and the controls did not reveal significant differences (Kruskal-Wallis rank sum test, *p* = 0.53).

**Figure 1:**
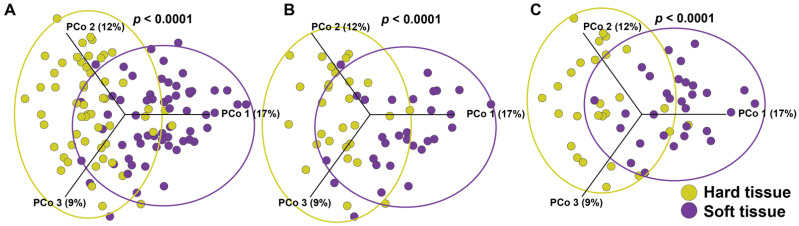
Beta diversity represented in Bray-Curtis Principal Component (PCo) plots (abundance) of bacterial communities in hard and soft tissue. (**A**) Samples from all participants (patients with PD and the controls) show statistically different microbiota communities for hard and soft tissues (Permanova, *p* < 0.0001). (**B**) Samples only from patients with PD show significant differences for hard and soft tissues (Permanova, *p* < 0.0001). (**C**) Samples only from the controls also show statistically different microbiota communities for hard and soft tissues (Permanova, *p* < 0.0001). Each point represents one sample; longer distances between points represent larger differences in microbiota composition.

We next assessed beta-diversity for hard versus soft tissue, using PERMANOVA to identify community differences in the presence or abundance of each species (Jaccard or Bray-Curtis, respectively) and differences in taxonomic lineage presence or abundance (Weighted UniFrac or Unweighted Unifrac, respectively). In our cohorts, hard and soft tissue niches harbor significantly different bacterial communities for all beta-diversity measures (*p* < 0.0001 for all statistical tests) (Bray-Curtis in Figure 1A). The distinction between hard and soft tissue remains intact both within patients with PD (Jaccard and Bray-Curtis *p* < 0.0001; Weighted UniFrac *p* = 0.003; and Unweighted UniFrac *p* = 0.002) and the controls (*p* < 0.001 for all statistical measures) (Bray-Curtis in Figure 1B,C for both groups). Using differential abundance analysis, 142 OTUs were found to be significantly different between hard and soft tissue samples. The 60 most abundant species are shown in Table S1.

### 3.3. Soft Tissue Community Abundance

**Figure 2:**
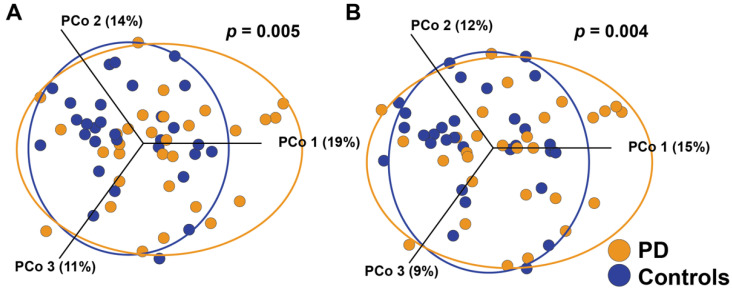
Difference in soft tissue microbiota composition in patients with PD and the controls. (**A**) Jaccard Principal Component (PCo) plot of bacterial communities shows a statistically significant difference of presence/absence of species between groups (Permanova, *p* = 0.004). (**B**) Bray-Curtis Principal Component (PCo) plot of bacterial communities also shows a statistically significant difference of abundance of species between groups (Permanova, *p* = 0.005). Each point represents one sample; longer distances between points represent larger differences in microbiota composition.

**Figure 3:**
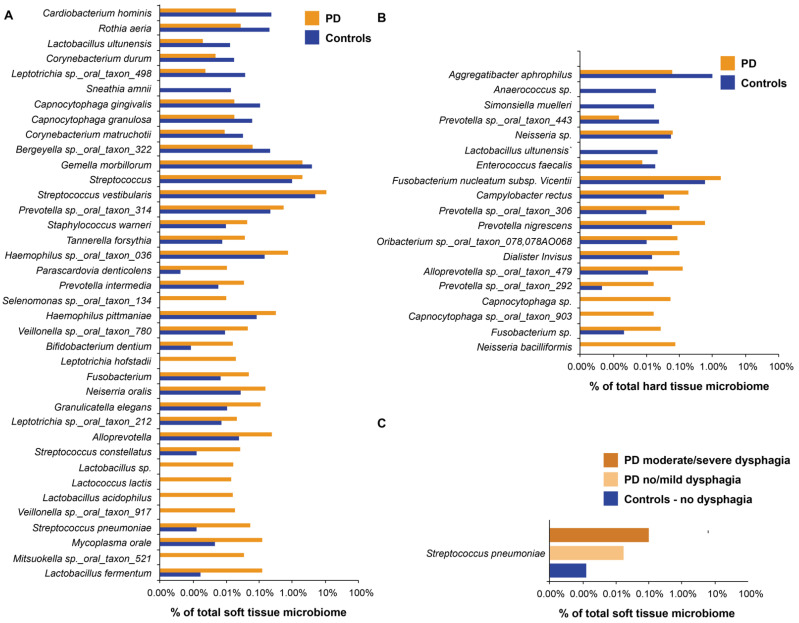
Top OTUs by relative differential abundance of species are statistically different between patients with PD and the controls. (**A**) Soft tissue. (**B**) Hard tissue. (**C**) Soft tissue, statistical differences in *S. pneumoniae* between no/mild and moderate/severe dysphagia in patients with PD. Controls have no dysphagia.

There were significant differences between the overall soft tissue microbiota in patients with PD and the controls, as assessed by OTU presence/absence and abundance (Figure 2; Figure S2 plots the same data on one and two axes) (Jaccard *p* = 0.004; Bray-Curtis *p* = 0.005, respectively). When the differential abundance was assessed between groups, 38 OTUs were found to be significantly different (Figure 3A). Soft tissue samples from patients with PD had a higher abundance of potential pathogenic oral species from the genera Lactobacillus, Tannerella forsythia, and Prevotella intermedia. Patients with PD also had increased abundance of endogenous opportunistic pathogens, such as Streptococcus pneumoniae, Mycoplasma orale, and Streptococcus constellatus.

### 3.4. Hard Tissue Community Abundance

We next focused on the hard tissue bacterial communities of patients with PD and the controls. As shown in Figure S3, there are no overall community differences (beta-diversity) in the hard tissue of patients with PD compared to the controls. We assessed the differential abundance between cohorts; 19 OTUs were found to be significantly different (Figure 3B). A total of 12 OTUs were significantly more abundant in patients with PD, and seven were significantly more abundant in the controls. More than fifty percent of the differentially expressed OTUs in patients with PD are possible opportunistic pathogens associated with gingivitis and mature plaque accumulation, consistent with the increased OHI-S values in this population (Fusobacterium, Capnocytophaga, Alloprevotella, Prevotella, and Campylobacter rectus) [24].

### 3.5. Disease Features with Impact on the Oral Microbiota in PD

To better understand why patients with PD have unique soft tissue bacteria, we analyzed the oral microbiota considering different factors that may affect oral health. When samples from patients with PD and the controls were grouped together, there were no significant differences in the oral microbiota composition by gender, age, race, ethnicity, BMI, level of education, and medications taken (i.e., non-antiparkinsonian medications) (Table 1). There were no significant differences when samples were analyzed by oral hygiene measures: oral health (BOHSE), plaque (OHI-S), times per day participants brushed or flossed their teeth, number of dental visits per year, or dietary habits (Table 2). Factors that significantly influenced soft tissue beta-diversity include dysphagia, drooling, and salivary pH (Table 2). Interestingly, *S. pneumoniae* was significantly increased, not only as a function of PD (Figure 3A), but also as a function of dysphagia (Figure 3C). Control subjects (no dysphagia) lacked this respiratory pathogen, whereas patients with no/mild (*n* = 17) or moderate/severe (*n* = 13) dysphagia had *S. pneumoniae* as 0.02% and 0.1% of their soft tissue microbiota, respectively (Figure 3C). Within patients with PD, those with mild disease severity (Hoehn and Yahr (H&Y) = 1–2) differed significantly from those with moderate to severe disease (H&Y = 3–5) on one of four measures of beta-diversity (Unweighted UniFrac *p* = 0.04) (Table 1). Those patients on levodopa/carbidopa had a significantly different oral microbiota than those not taking these medications (Jaccard *p* = 0.04; Bray-Curtis *p* = 0.03) (Table 1). Patients on levodopa/carbidopa medication showed a similar beta-diversity to the controls.

## 4. Discussion

The objective of this study was to characterize the oral microbiota among patients with PD who have different oral-related symptoms than the controls. Alpha-diversity measures did not reveal significant differences in community richness between patients with PD and the controls. Beta-diversity significantly differed between hard and soft tissue in both patients with PD and the controls. These results suggest that the oral environment in PD supports a microbial community that is normal in overall diversity and community abundance, and the distinctions between hard and soft tissue sites as niches are maintained. This is in agreement with previous reports [9,10].

We determined oral health by visual assessment of both hard and soft tissues and, in agreement with previous studies, found that patients with PD have worse oral health than age-, gender-, and education-matched controls [4]. Although overall hard tissue community abundance did not differ between the groups, the abundance of individual OTUs associated with increased plaque accumulation was greater in patients with PD. Therefore, the abundance of particular plaque and gingivitis associated bacteria was greater in PD, consistent with their elevated BOHSE (oral health) and OHI-S (plaque) scores. One limitation of our study is that we did not measure periodontitis status. Periodontitis and oral dysbiosis are known to be correlated, but it is possible that periodontitis shows a correlation with oral dysbiosis not seen when only measuring plaque index. Future studies should compare how periodontitis status may further influence the oral microbiota of these patients and how dysphagia and periodontitis interact to influence risk for aspiration pneumonia.

We found that oral hygiene habits did not differ significantly between the two groups, suggesting that this is not a factor influencing the poor oral health of these patients. This is in contrast with a study by Müller et al. that found lower frequency of toothbrushing and longer time since the last visit to the dentist for patients with PD [25]. We maintain that our results differ as we controlled for education level, a measure known to affect oral hygiene and health [26]. Despite similar frequency of oral hygiene in our study, patients with PD could have poor oral health due to their movement disorder reducing effectiveness of toothbrushing. This is not the case, however, as patients’ plaque index score improved significantly after toothbrushing and matched that of the controls. Taken together, this supports the hypothesis that poor oral health and oral dysbiosis in PD is not due to reduced frequency or efficiency of oral hygiene, but rather is related to disease-specific factors that result in increased abundance of OTUs known to incite oral disease. Our data supporting this idea that the pathogenesis of PD involves the oral cavity corroborates recent work [11].

In contrast to hard tissue, soft tissue beta-diversity was significantly different between PD and control groups. Analysis of individual OTUs demonstrates that patients with PD had higher abundance of species associated with opportunistic oral and respiratory disease. *S. pneumoniae* is found more commonly in our PD subjects, and is a common pathogen in aspiration pneumonia. Consistent with other studies, our patients also have a significant increase in multiple *Lactobacillus* species with functions in PD that may advance the disease [10]. Our results suggest that patients with PD have an oral microbiota with higher abundance of harmful bacteria compared to the controls.

Diet is a factor known to influence oral health and microbiota [27]. In this study, patients with PD reported consuming more snacks and sugary drinks per day than the controls. However, these factors did not seem to influence oral microbiota composition significantly. Future studies are needed to understand how a liquid and/or high sugar diet may influence the microbiota of patients with PD. Dysphagia and drooling problems may be main factors responsible for the deterioration of oral health in patients with PD [28,29]. Indeed, our patients with PD showed significantly worse dysphagia and drooling than the controls, and PD subjects with dysphagia were more likely to harbor *S. pneumoniae*. In agreement with previous studies, our results show that salivary pH significantly influences microbiota composition in both patients with PD and the controls [27]. Prior work established that gut microbiota change in patients taking anti-parkinsonian medications [30]. Our results show that these medications may also influence the composition of the oral microbiota, shifting beta-diversity towards health. Further studies are needed to better understand how PD medication may influence the oral microbiota and overall oral health. Finally, our results show a small but significant difference in beta-diversity between patients with mild and moderate/severe PD measured by H&Y. Future studies should further explore changes in the oral microbiota across time and disease severity.

The study has some limitations. Dysphagia was measured with a subjective method (questionnaire), whereas objective methods such as a video-fluoroscopy swallow test can show alterations in swallowing even in patients without subjective complaint [31]. As objective methods are invasive and expensive, a validated questionnaire is an acceptable alternative in our study. We found a significant difference in the oral microbiota between patients with PD taking levadopa/carbidopa and those not taking them. Patients taking these medications showed similar beta-diversity to the controls. As a within-patient analysis results in a low sample size, future studies are needed to fully investigate how PD medication may affect the oral health and microbiota of these patients. Although we controlled for sugar and carbohydrate intake, there may be other dietary differences, such as probiotic consumption, that could affect oral microbiota. Finally, as a cross-sectional design, this study does not characterize how the microbiota, dysphagia severity, and other health and behavioral factors may change with time. Future studies addressing these questions will help better understand if changes in oral health are a result or consequence of PD and/or its symptoms.

Our findings suggest that oral-related symptoms of PD may influence the oral microbiota and account for some of the differences observed when compared to control subjects. Future work may lead to the development of diagnostic and/or treatment strategies to improve the quality of life and extend survivability of patients with PD. By preventing aspiration pneumonia, the main cause of death in this patient population, the oral microbiota may prove to be a noninvasive, inexpensive, and life-saving biomarker.

## Figures and Tables

**Table 1:** Demographics and clinical information.

	PD (*n* = 30)	Controls (*n* = 30)	*p*-Value ^†^	Jaccard *p*-Value	Bray-Curtis *p*-Value	Weighted Unifrac *p*-Value	Unweighted Unifrac *p*-Value
**Gender (%)**			0.43	0.97	0.97	0.81	0.63
Female	37	43					
Male	63	57					
**Age (years, mean ± SD)**	69.2 ± 9.4	69.1 ± 8.4	0.94	0.76	0.76	0.67	0.80
**Race/Ethnicity (%)**			0.13	0.06	0.10	0.11	0.46
Black	17	17					
Hispanic/Latino	10	20					
White	73	63					
**Education (%)**			0.42	0.85	0.86	0.94	0.37
≤High school diploma	27	33.3					
Some college/Associates degree	23	33.3					
≥Bachelor’s degree	50	33.3					
**BMI (mean ± SD)**	27 ± 6.0	28.6 ± 6.6	0.33	0.8	0.8	0.9	0.5
**PD Medications (%)**				0.04 ^‡,^*	0.03 ^‡,^*	0.36 ^‡^	0.66 ^‡^
Levadopa and/or Carbidopa	63	0					
COMT inhibitor	20	0					
Dopamine agonist	13	0					
MAO inhibitor	17	0					
Aticholinergic	3	0					
None	30	100					
**Other medications (%)**							
Aspirin	20	43	0.03 *	0.21	0.27	0.25	0.10
Statins (heart disease)	13	30	0.12	0.48	0.54	0.25	0.23
Metformin (diabetes)	6	23	0.07	0.16	0.17	0.49	0.05
Levothyroxine sodium (thyroid)	3	23	0.02 *	0.52	0.48	0.60	0.89

PD: Parkinson’s disease; BMI: body mass index; COMT: catechol-O-methyl transferase; and MAO: monoamine oxidase. ^†^ Chi-square calculated for categorical data and Student’s *t*-test for continuous data, ^‡^ Analysis performed only within patients with PD, * *p* < 0.05.

**Table 2:** Oral health and hygiene.

	PD (*n* = 30)	Controls (*n* = 30)	*p*-Value ^†^	Jaccard *p*-Value	Bray-Curtis *p*-Value	Weighted Unifrac *p*-Value	Unweighted Unifrac *p*-Value
**Total BOHSE** **(mean ± SD)**	4.61 ± 0.5	2.97 ± 0.5	0.02 *	0.14	0.17	0.03 *	0.31
**OHI-S (mean ± SD)**							
Before TB	1.6 ± 0.13	1.0 ± 0.16	0.006 *	0.84	0.83	0.94	0.19
After TB	0.54 ± 0.08	0.39 ± 0.11	0.29	0.53	0.45	0.84	0.16
Total Change	1 ± 0.1	0.62 ± 0.1	0.01 *	0.85	0.90	0.99	0.12
**ROMP (mean ± SD)**							
Swallowing score	11.5 ± 1.0	7.0 ± 0.03	<0.001 *	0.02 *	0.02 *	0.04 *	0.19
Saliva score	15.8 ± 1.3	9.2 ± 0.1	<0.001 *	0.19	0.17	0.02 *	0.03 *
**Salivary pH** **(mean ± SD)**	6.7 ± 0.19	7.1 ± 0.16	0.08	0.001 *	<0.001 *	0.001 *	0.004 *
**Oral hygiene (%)**							
TB ≥ 2X/day	60	73	0.27	0.69	0.68	0.71	0.69
Flosses ≥ 1X/day	47	50	0.80	0.21	0.28	0.42	0.14
Dental visits ≥ 2X/year	57	50	0.60	0.12	0.16	0.09	0.12
**Diet (%)**							
Eats ≥ 4 snacks/day	23	3	0.02 *	0.12	0.13	0.13	0.15
Never eats foods with sugar added	13	40	0.02 *	0.55	0.58	0.55	0.27
Never sugary drinks	33	20	0.24	0.11	0.13	0.27	0.07

PD: Parkinson’s disease; BOHSE: Brief Oral Health Status Examination; OHI-S: Simplified Oral Hygiene Index; TB: toothbrushing; and ROMP: Radboud Oral Motor Inventory. ^†^ Chi-square calculated for categorical data and Student’s *t*-test for continuous data, * *p* < 0.05.

## Data Availability

Data is contained within the article and supplementary material.

## Supplementary Materials

The following are available online at https://www.mdpi.com/article/10.3390/microorganisms9081616/s1, Table S1: Most abundant species that differ by hard and soft tissue, Figure S1: Microbiota richness and diversity comparisons between hard and soft tissue communities, Figure S2: Bray-Curtis Principal Component plots of bacterial communities in patients with PD and controls, and Figure S3: Beta diversity of hard tissue.
