# Supplementary material for: Oral Factors That Impact the Oral Microbiota in Parkinson’s Disease
Source: Microorganisms. 2021 Jul 29;9(8):1616. doi: 10.3390/microorganisms9081616 (PMC8402101; doi:10.3390/microorganisms9081616)
Supplement: Supplementary file 1 [file microorganisms-09-01616-s001.zip › microorganisms-1285682-supplementary.pdf]

Oral factors that impact the oral microbiota in Parkinson's disease

Natalia S. Rozas, Ph.D., Gena D. Tribble, Ph.D. and Cameron B. Jeter, Ph.D.

SUPPLEMENTAL FIGURES

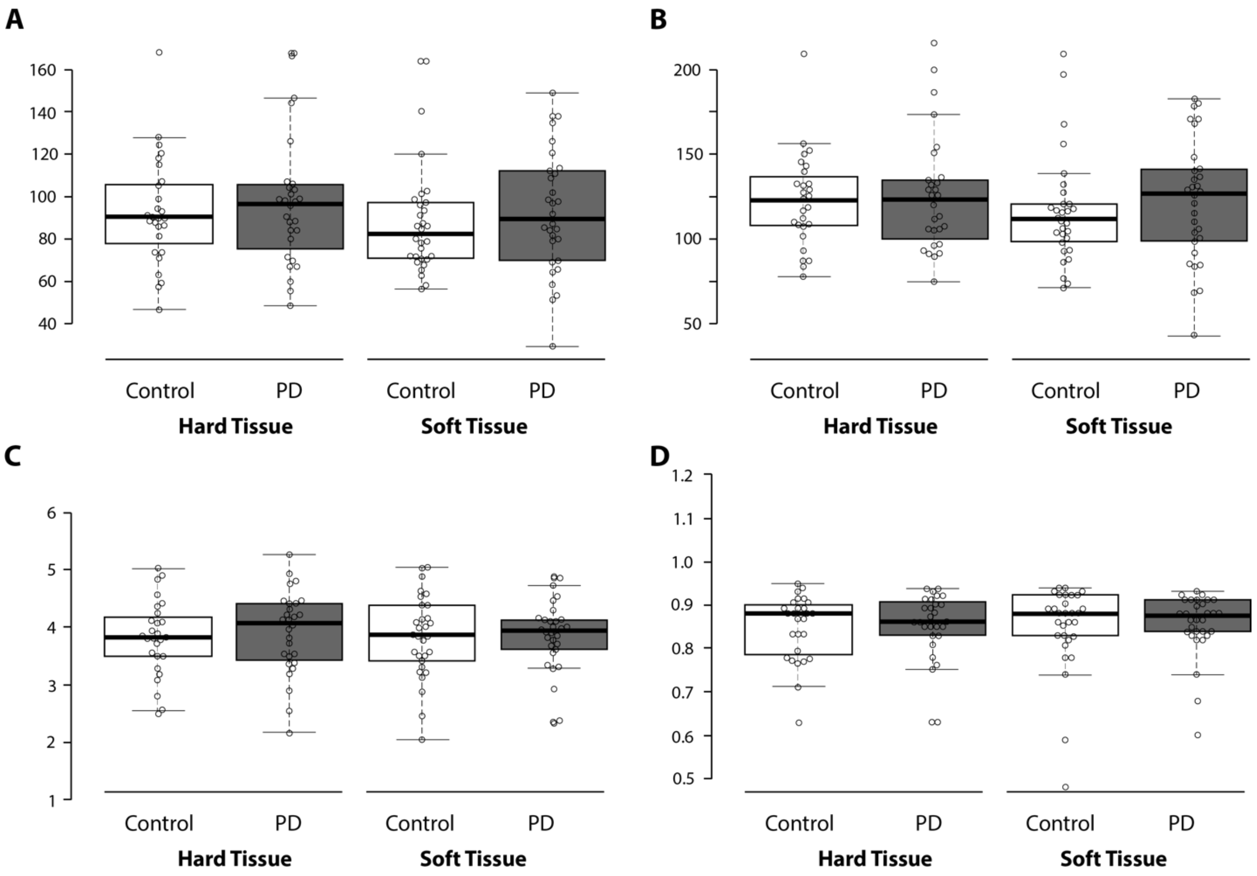

Figure S1: Microbiota richness and diversity comparisons between hard and soft tissue communities. **A)** Total OTUs and **B)** Chao-1 values are shown for sample richness in control's hard tissue (n=27), soft tissue (n=30) and PD's hard tissue (n=28) and soft tissue (n=30). **C)** Shannon entropy and **D)** Simpson's Index of Diversity (1-D) represent diversity and evenness estimates. There are no significant differences in richness or diversity between sample sites or subject cohorts, as assessed by Kruskal-Wallis ANOVA. Box limits indicate the 25th and 75th percentiles as determined by R software; center lines

show medians; whiskers extend 1.5 times the interquartile range from the 25th and 75th percentiles; data points are plotted as open circles.

	Hard Tissue		Soft Tissue			Hard Tissue		Soft Tissue	
Taxonomy	Healthy control	Parkinson's disease	Healthy control	Parkinson's disease	Taxonomy	Healthy control	Parkinson's disease	Healthy control	Parkinson's disease
Haemophilus parainfluenzae	20.00%	11.00%	22.00%	13.00%	*Haemophilus sp._oral_taxon_036*	0.51%	0.81%	0.15%	0.76%
Streptococcus sp._oral_taxon_058	19.00%	21.00%	10.00%	16.00%	*Fusobacterium nucleatum_subsp._vincentii*	0.50%	2.00%	0.14%	0.52%
Neisseria flava	7.00%	4.00%	0.43%	0.70%	***Bergeyella sp._oral_taxon_322***	0.49%	0.79%	0.21%	0.06%
Streptococcus sanguinis	5.00%	5.00%	0.65%	0.49%	***Neisseria oralis***	0.46%	0.48%	0.03%	0.16%
Rothia dentocariosa	4.00%	8.00%	0.59%	0.91%	***Actinomyces sp._oral_taxon_180***	0.46%	0.18%	2.00%	0.93%
Streptococcus sp._oral_taxon_057	3.00%	2.00%	6.00%	7.00%	***Actinomyces sp._oral_taxon_170***	0.44%	0.38%	0.04%	0.03%
Gemella morbillorum	3.00%	2.00%	4.00%	2.00%	*Haemophilus paraphrohaemolyticus*	0.39%	0.27%	0.31%	0.35%
Lautropia mirabilis	3.00%	2.00%	0.06%	0.08%	***Kingella sp._oral_taxon_012***	0.37%	0.41%	0.04%	0.02%
Streptococcus vestibularis	2.00%	4.00%	5.00%	11.00%	***Fusobacterium periodonticum***	0.37%	0.54%	3.00%	1.00%
Neisseria subflava	2.00%	2.00%	8.00%	6.00%	***Aggregatibacter sp._oral_taxon_513***	0.33%	0.11%	0.05%	0.01%
Streptococcus sp._oral_taxon_056	2.00%	2.00%	1.00%	2.00%	***Kingella oralis***	0.31%	0.55%	0.03%	0.05%
Streptococcus cristatus	2.00%	2.00%	1.00%	2.00%	***Capnocytophaga granulosa***	0.24%	0.29%	0.06%	0.02%
Actinomyces sp._oral_taxon_169	2.00%	1.00%	0.25%	0.26%	***Eikenella corrodens***	0.23%	0.43%	0.03%	0.01%
Granulicatella adiacens	1.00%	0.79%	3.00%	2.00%	***Streptococcus mutans***	0.21%	0.93%	0.03%	0.19%
Streptococcus sp._oral_taxon_074	1.00%	0.69%	3.00%	1.00%	*Fusobacterium nucleatum_subsp._animalis*	0.17%	0.73%	0.14%	0.28%
Rothia aeria	1.00%	0.60%	0.20%	0.03%	*Prevotella oris*	0.14%	0.50%	0.07%	0.28%
Veillonella dispar	1.00%	2.00%	4.00%	5.00%	***Prevotella sp._oral_taxon_314***	0.11%	0.23%	0.22%	0.54%
Aggregatibacter sp._oral_taxon_458	1.00%	2.00%	0.17%	0.29%	***Veillonella rogosae***	0.09%	0.08%	0.44%	0.36%
Kingella denitrificans	1.00%	3.00%	0.06%	0.06%	*Haemophilus pittmaniae*	0.09%	0.28%	0.08%	0.32%
Aggregatibacter aphrophilus	1.00%	0.05%	0.06%	0.02%	***Campylobacter concisus***	0.08%	0.11%	0.36%	0.34%
Veillonella parvula	0.95%	0.53%	0.83%	0.99%	***Leptotrichia sp._oral_taxon_417***	0.07%	0.07%	0.54%	0.31%
Cardiobacterium hominis	0.89%	0.51%	0.22%	0.02%	*Prevotella nigrescens*	0.05%	0.48%	0.06%	0.25%
Abiotrophia defectiva	0.87%	0.41%	0.08%	0.03%	***Actinomyces graevenitzii***	0.04%	0.02%	0.58%	0.32%
Capnocytophaga gingivalis	0.84%	0.38%	0.10%	0.02%	***Prevotella sp._oral_taxon_306***	0.01%	0.10%	0.43%	0.52%
Porphyromonas sp._oral_taxon_278	0.74%	1.00%	1.00%	0.94%	***Prevotella histicola***	0.10%	0.28%	2.00%	3.00%
Capnocytophaga sputigena	0.59%	0.29%	0.03%	0.01%	***Actinomyces sp._oral_taxon_172***	0.08%	0.06%	0.88%	0.55%
Fusobacterium nucleatum_subsp._polymorphum	0.57%	2.00%	0.11%	0.22%	***Prevotella sp._oral_taxon_313***	0.07%	0.14%	0.72%	1.00%
Prevotella melaninogenica	0.54%	1.00%	5.00%	8.00%	***Prevotella salivae***	0.05%	0.16%	0.81%	1.00%
Capnocytophaga leadbetteri	0.53%	0.95%	0.06%	0.03%	***Prevotella nanceiensis***	0.04%	0.16%	1.00%	0.49%

Table S1. Most abundant species that differ by hard and soft tissue. 142 OTUs were found to be differentially abundant between sampling sites of hard and soft tissue. OTUs that are significantly different between hard and soft tissue are shown in bold on this list of most abundant 60 species.

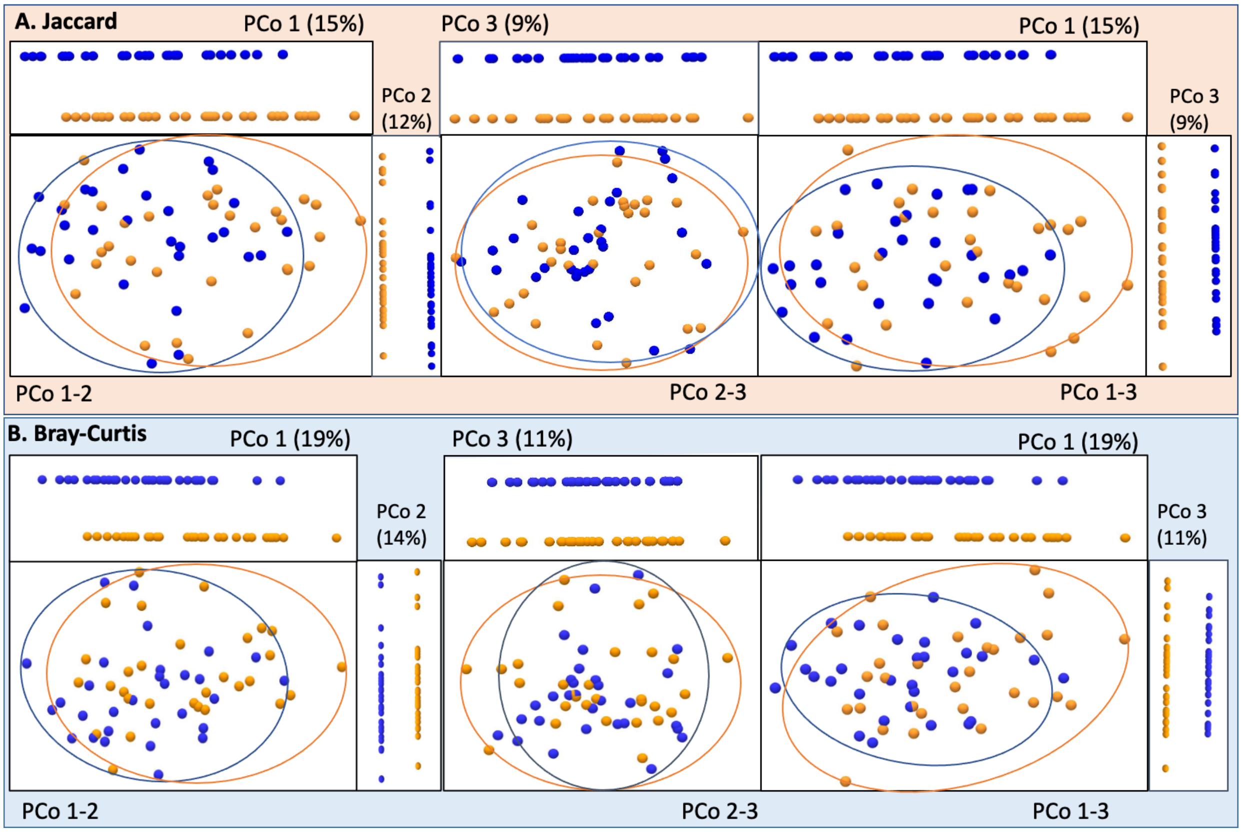

Figure S2: (A) Jaccard and (B) Bray-Curtis Principal Component (PCo) plots of bacterial communities in patients with Parkinson's disease (orange) and controls (blue). For each panel, one dimensional comparisons are shown on the X (PCo 1, PCo 3) and Y (PCo2, PCo 3) axes. Two dimensional plots for PCo 1-2, PCo 2-3, and PCo 1-3 are shown from left to right. Both Jaccard and Bray-Curtis PERMANOVA values are significant, with *p*-values of 0.004 and 0.005, respectively. This figure is supplemental to Figure 2, which shows community distribution in 3 dimensions (PCo 1,2,3).

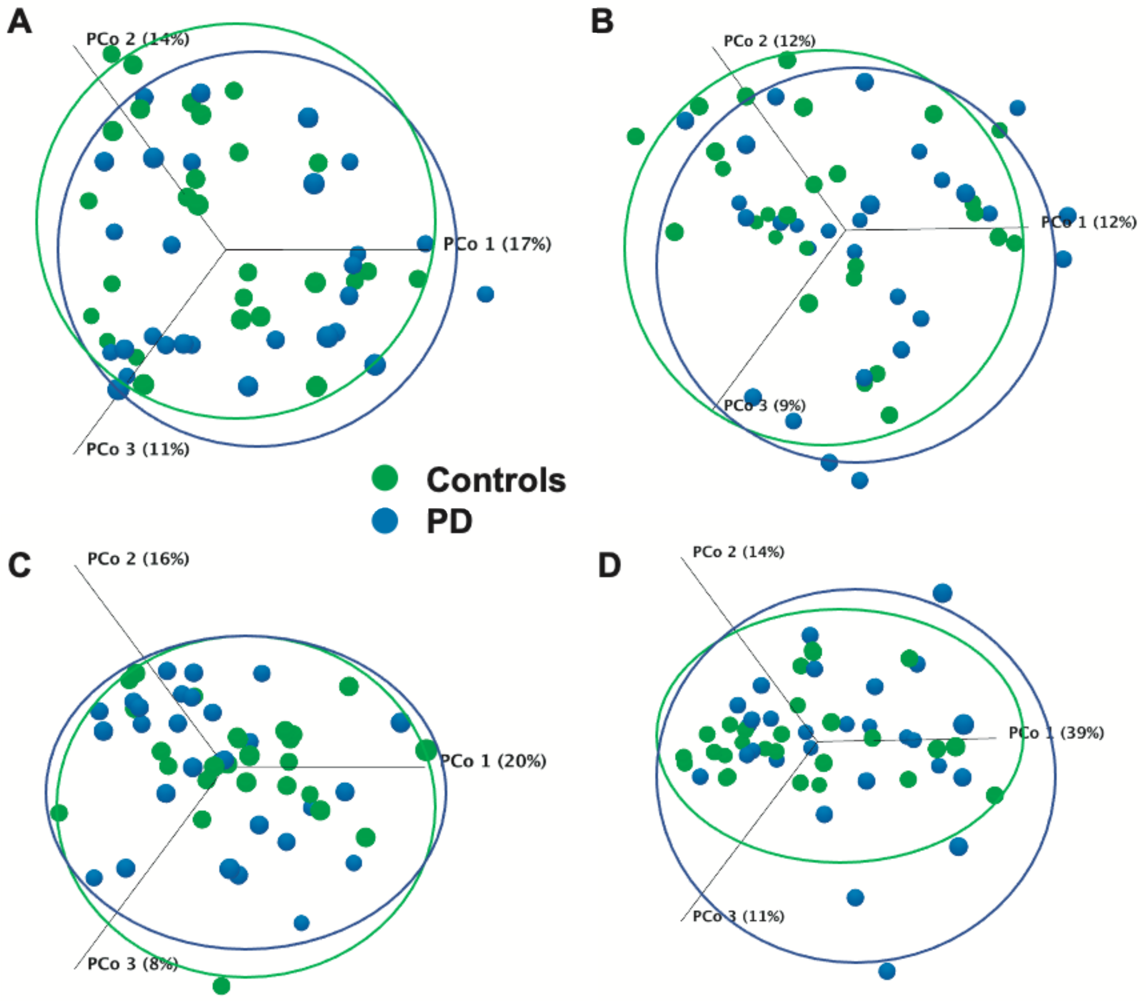

Figure S3: Beta diversity of hard tissue. There were no significant differences in hard tissue microbiota composition between patients with PD (blue) and controls (green). Community distributions are indicated with colored circles. **A)** Bray-Curtis Principal component (PCo) plot (Permanova, p = 0.246). **B)** Jaccard Principal component (PCo) plot (Permanova, p = 0.237). **C)** Weighted UniFrac Principal component (PCo) plot (Permanova, p = 0.136). **D)** UnWeighted UniFrac Principal component (PCo) plot (Permanova, p = 0.27). Each point represents one sample; longer distances between points represent larger differences in microbiota composition.
